# Caregiving burden and depression in paid caregivers of hospitalized patients: a pilot study in China

**DOI:** 10.1186/s12889-017-4563-6

**Published:** 2017-07-25

**Authors:** Yao-Dan Liang, Ya-Li Wang, Zhi Li, Li He, Ying Xu, Qing Zhang, Gui-Ying You, Xu-Hua Mi

**Affiliations:** 1 Department of Cardiology, West China Hospital, Sichuan University, Chengdu, Sichuan China; 2 Department of Nephrology, West China Hospital, Sichuan University, No. 37, Guo Xue Xiang, Chengdu, Sichuan 610041 China

**Keywords:** Caregiver burden inventory, Depression, Paid caregiver, China

## Abstract

**Background:**

Caregiving burden and depression in family caregivers have been investigated, but little is known about how they affect paid caregivers. The aim of this study was to investigate caregiving burden and depression in paid caregivers of hospitalized patients.

**Methods:**

A cross-sectional survey study was conducted in a tertiary referral hospital (Chengdu, China) that enrolled 108 paid caregivers who worked in the inpatient department. The Caregiver Burden Inventory (CBI) and the Center for Epidemiologic Studies Depression (CES-D) scale were incorporated into a self-developed questionnaire to gather demographic information on the following four aspects: general, work, income, and family.

**Results:**

The mean total CBI score was 29.7 ± 12.5. The time-dependence burden had the highest score of 15.3 ± 4.0, which was followed by the physical burden score of 6.5 ± 4.6, developmental burden score of 3.7 ± 4.0, social burden score of 3.2 ± 4.0, and emotional burden score of 2.4 ± 3.1. Multiple linear regression analysis showed that a higher CBI was associated with a longer time as a paid caregiver [β=7.041, 95% Confidence Interval (CI):1.935 to 12.974, *p* = 0.009], lower income satisfaction (β= − 6.573, 95% CI: -11.248 to −3.020, *p* = 0.001), and higher frequency of meeting with their relatives (β=7.125, 95% CI: 2.019 to 12.456, *p* = 0.006). The mean CES-D score was 11.9 ± 8.7, and significant depression was found in 28 (25.9%) paid caregivers according to the CES-D score ≥ 16 cut-off. There was a moderate positive correlation between the CBI and CES-D scores (Pearson’s *r* = 0.452, *p* < 0.001).

**Conclusions:**

A high caregiving burden was commonly observed in paid caregivers of hospitalized patients in China, as was a high prevalence of depression symptoms. Several associated factors were identified that could be areas for future interventions.

## Background

Caregivers play a pivotal role in patient-centred care because they counteract the deficit of professional nurses in many countries. [1–3] In particular, the number of paid caregivers, who serve as a replacement or supplement for nurses, has been rapidly increasing. [4–6] Caregivers have taken on the majority of basic care, including making the bed, feeding and bathing the patient, cleaning urine and excrement, and helping with expectoration. In addition, they can administer medication, provide information, facilitate communication and provide emotional support. Although there are strong family traditions and an understood filial responsibility to provide care in China, family members who act as caregivers are limited in the time they can spend caring for patients outside of work. Therefore, caregiver companies have gradually flourished, and they are authorized by hospitals to provide a continuous supply of paid caregivers. Per regulations, paid caregivers are registered with a caregiver company, from which they are hired by patients/relatives for temporarily contracted caregiving work.

Caregivers have been shown to suffer from high caregiving burden and depression; therefore, they are a group of vulnerable people or “invisible patients” who need attention or interventions from medical staff and social workers. [7, 8] However, most previous studies [1, 7, 8] have focused on the caregiving burden of family caregivers for patients who stayed at home and not on the growing population of paid caregivers who work in hospitals. According to published studies in Chinese journals, most paid caregivers are middle-aged women who were previously farmers from a rural area or laid-off workers from a city. [9, 10] Although psychological health and work pressure were investigated in paid caregivers in China, which were related to sleep disturbances, unstable income, and a lack of social respect and support, [11, 12] data are very limited on the caregiving burden in this population, especially by the use of designated inventories. Therefore, to provide information for developing practical interventions, a survey study was conducted to identify the prevalence of caregiving burden and depression symptoms as well as their associated factors from four categories, including general, work, income, and family characteristics.

## Methods

### Study design

This was a cross-sectional survey study in a tertiary referral hospital (Chengdu, China) performed with a self-developed questionnaire that is described in the “Instruments” section. Nurses participated in the design, distribution and return of the questionnaire.

### Participants

Considering that caregiving burden could be affected by the working environment and patients of paid caregivers, the subjects were all paid caregivers who worked in four medical and four surgical wards (i.e., cardiology vs. cardiac surgery, respiratory medicine vs. thoracic surgery, gastrointestinal medicine vs. gastrointestinal surgery, and neurology vs. neurosurgery). The subjects were enrolled if they met the following criteria: (1) agreed to participate and signed the informed consent form and (2) could read and complete the questionnaire. Consequently, 120 of the 124 (96.8%) registered paid caregivers from the aforementioned wards were enrolled; four people did not fulfil the inclusion criteria as they refused to participate.

### Instruments

The questionnaire was written in Chinese and consisted of three parts. In the first part, the baseline characteristics of the study subjects were collected; this included proposed factors associated with the caregiving burden or depression that were categorized into general, work, income, and family information. General information included the age, gender, education, and marital status. Work information included the ward in which they worked, number of years as a paid caregiver, and number of vacation days per month. For the income information, “income satisfaction” was a required field, while “income per month before and after becoming a paid caregiver” was optional. The number of dependents, frequency of meeting his/her own family, and housing status in Chengdu (owned or rental) were categorized as family information.

**Fig. 1:**
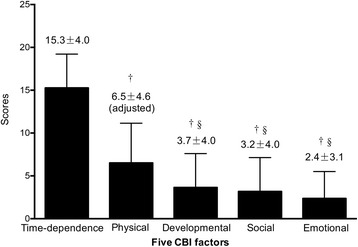
Five CBI factors and their scores. CBI, Caregiving Burden Inventory. The bar chart represents the means ± standard deviation, and the adjusted physical burden is the original score multiplied by 1.25 to give an equivalent score out of 20 for comparison. †*p* < 0.001 vs. the time-dependence burden and §*p* < 0.001 vs. the physical burden

The second part assessed the caregiving burden by adopting the Chinese version of the Caregiver Burden Inventory (CBI), which has been validated and shown to be sufficiently reliable in a Chinese population. [13, 14] The CBI has 24 items, and each item is answered on a scale ranging from 0 (not at all disruptive) to 4 (very disruptive). The CBI describes the caregiving burden in 5 different domains (factors): time-dependence (items 1 to 5), developmental (items 6 to 10), physical (items 11 to 14), social (items 15 to 19), and emotional (items 20 to 24) burden. The time-dependence burden is associated with the time restrictions of caregivers. The developmental burden describes the perception of being “taken out” of their development or being excluded from the expectations and opportunities enjoyed by their peers. The physical burden is associated with feelings of chronic fatigue and their own physical health problems. The social burden describes the poor balance between many roles. The emotional burden is associated with negative feelings towards patients and is possibly induced by the unpredictable and bizarre behaviours of the patients. [15, 16] Consequently, the score in the physical burden factor should range from 0 to 16 (only 4 items), and each of the scores in the other factors should range from 0 to 20 (5 items for each factor). The total score of the CBI ranges from 0 to 96, and a higher score indicates a higher caregiving burden. To compare between the 5 aforementioned factors (Fig. 1), the physical burden score was multiplied by 1.25 to give an equivalent score out of 20. [15].

The third part of the questionnaire assessed the depression symptoms of paid caregivers with the Chinese version of the Center for Epidemiologic Studies Depression Scale (CES-D), which has been validated and showed good reliability in a Chinese population. [17–19] The CES-D includes 20 items that cover affective, cognitive, behavioural, and somatic symptoms associated with depression. The subjects were asked to indicate the frequency of symptoms on the following 4-point scale from 0 (rarely or none of the time) to 3 (most or all of the time). The 4 positively formulated items (items 4, 8, 12, and 16) were recorded in reverse. Therefore, the total score of the CES-D ranged from 0 to 60, and a score of 16 or higher indicates the likely presence of clinically significant depression. [18, 20].

According to the study design, the copies of the questionnaire with inadequate quality were not entered into the final analysis, including those with an incomplete CBI or CES-D section, and a consistent lowest score (i.e. “0” score) or highest score (i.e. “4” score in the CBI section and “3” score in the CES-D section) for each item in either section. This extreme and unchangeable scoring situation was considered to indicate that the participant had not understood the survey or had not seriously considered it.

### Statistics

The statistical analysis was performed using SPSS software, version 20.0 (SPSS, Chicago, IL, USA). A Kolmogorov-Smirnov test was performed to determine whether continuous parameters were normally distributed. T-tests, chi-square tests and Fisher’s exact tests were used for univariate analyses. One-way ANOVA was used to test differences among the 5 factors of the CBI. Multiple linear regression was performed to identify factors associated with the CBI and CES-D scores. A Pearson correlation coefficient was used to analyse the correlations between the CBI and CES-D scores. A *p*-value <0.05 was considered statistically significant.

## Results

During the study period, 120 copies of the survey were sent out and returned (100% response rate) by assigned ward nurses. However, several surveys were unsuitable for analysis (12/120, 10%), including nine that had an incomplete CBI or CES-D section and another three that had a consistent “0” score for each item in the CBI section.

### Baseline characteristics

Data for the 4 domains are presented in Table 1. All paid caregivers were middle-aged, and most were female. The majority of the subjects achieved an educational level below high school. The number of years that each participant worked as a paid caregiver was categorized into five groups in the questionnaire (i.e., less than 1 year, 1 ~ 3 years, 3 ~ 5 years, 5 ~ 10 years, and more than 10 years); however, 73 (66.7%) paid caregivers worked in this field for more than 10 years.

**Table 1:** Baseline characteristics of the paid caregivers

Characteristics	Paid caregivers (*n* = 108)
General	
Age (y), mean ± SD	47.6 ± 5.1
Gender, n (%)	
Male	34 (31.5)
Female	74 (68.5)
Education, n (%)	
Primary school or lower	38 (35.2)
Middle school	54 (50.0)
High school or higher	16 (14.8)
Work	
Ward in which they worked, n (%)	
Medical	45 (41.7)
Surgical	63 (58.3)
Time as a paid caregiver, n (%)	
≥ 10 years	72 (66.7)
< 10 years	36 (33.3)
Vacation days per month, n (%)	
≤ 4 days	83 (76.9)
> 4 days	25 (23.1)
Income	
Income satisfaction, n (%)	
Satisfied	58 (53.7)
It’s OK	40 (37.0)
Unsatisfied	10 (9.3)
Family	
Number of dependents, n (%)	
≤ 2	29 (26.9)
3 ~ 4	39 (36.1)
≥ 5	40 (37.0)
Frequency of meeting with family, n (%)	
≥ 1 in every month	50 (46.3)
< 1 in every month	58 (53.7)
Housing status in Chengdu, n (%)	
Yes	57 (52.8)
No	51 (47.2)

n, the number of subjects; SD, Standard Deviation

### Caregiving burden and depression scores in paid caregivers

The total CBI score was normally distributed among the study population with a mean value of 29.7 ± 12.5. There were some differences among the 5 factors of the CBI that were detected by the ANOVA (F _(4535)_ = 194.207, *P* < 0.001) and post hoc analysis. The time-dependence burden factor had the highest score of 15.3 among the 5 factors; it was significantly higher than those of the remaining factors (all *P* < 0.001). It was followed by the physical burden score of 6.5, which was significantly higher than the emotional, developmental, and social burden scores (all *P* < 0.001). The scores of the last 3 factors were similar. (Fig. 1).

**Fig. 2:**
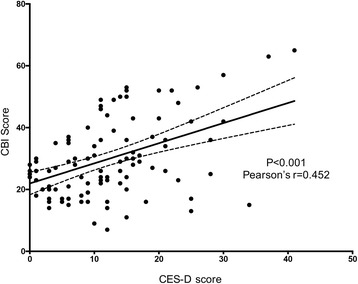
Correlation between the CES-D and CBI scores. CES-D, Center for Epidemiologic Studies Depression Scale. CBI, Caregiving Burden Inventory. The Pearson correlation coefficient is 0.452 (*P* value <0.001)

The CES-D score of the study participants also had a normal distribution with a mean value of 11.9 ± 8.7. Using the CES-D score ≥ 16 cut-off, depression was found in 28 (25.9%) paid caregivers. Moreover, the CBI score of the depression group (CES-D score ≥ 16) was significantly higher than that of the non-depression group (36.3 ± 14.3 vs. 27.4 ± 11.0, *P* = 0.001). The CBI score was positively correlated with the CES-D score (*r* = 0.452, *P* < 0.001). (Fig. 2).

### Factors associated with a higher caregiving burden and depression

A multiple linear regression analysis was used to identify the factors that were related to higher CBI (Table 2) and CES-D (Table 3) scores. The parameters in Table 1 were all included as covariates in the model, where either the CBI or CES-D score was the dependent variable. Three independent variables were associated with a higher CBI score, which included a longer time as a paid caregiver (*P* = 0.009), lower income satisfaction (*P* = 0.001), and more frequent meeting with his/her own family (*P* = 0.006). (Table 2) Similarly, a lower income satisfaction was found to be associated with a higher CES-D score (*P* = 0.008). (Table 3).

**Table 2:** Variables associated with the likelihood of a higher CBI score

Variables	Unstandardized Coefficients		Standardized Coefficients	t	95% CI for β		*P* values
	*β*	SE	Beta		Lower	Upper	
Age	0.167	0.265	0.069	0.630	−0.380	0.718	0.530
Gender	4.872	2.808	0.182	1.735	−0.859	10.800	0.086
Education level	3.379	1.839	0.184	1.838	−0.362	7.270	0.069
Ward in which they worked	−1.713	2.528	−0.068	−0.678	−6.619	3.874	0.500
Time as a paid caregiver	7.041	2.659	0.268	2.648	1.935	12.974	0.009
Vacation days per month	−3.942	2.819	−0.134	−1.398	−9.680	2.022	0.165
Number of dependents	−0.700	1.693	−0.045	−0.413	−4.233	2.794	0.680
Frequency of meeting with family	7.125	2.514	0.286	2.834	2.019	12.456	0.006
Housing status in Chengdu	−0.432	2.412	−0.017	−0.179	−4.617	5.397	0.858
Income satisfaction	−6.573	1.982	−0.348	−3.316	−11.248	−3.020	0.001
Constant	9.785	18.041		0.542	−26.851	48.043	0.589

*SE* standard error, *CI* confidence interval

**Table 3:** Variables associated with the likelihood of a higher CES-D score

Variables	Unstandardized Coefficients		Standardized Coefficients	t	95% CI for β		*P* values
	β	SE	Beta		Lower	Upper	
Age	0.000	0.186	0.000	0.002	−0.366	0.367	0.999
Gender	0.916	1.960	0.049	0.467	−2.974	4.806	0.641
Education level	−0.921	1.283	−0.072	−0.718	−3.468	1.626	0.475
Ward in which they worked	−3.429	1.764	−0.196	−1.944	−6.930	0.072	0.055
Time as a paid caregiver	0.939	1.856	0.051	0.506	2.745	4.622	0.614
Vacation days per month	−0.990	1.967	−0.048	−0.503	−4.894	2.915	0.616
Number of dependents	−1.652	1.181	−0.152	−1.398	−3.997	0.693	0.165
Frequency of meeting with family	0.513	1.755	0.030	0.292	2.969	3.995	0.771
Housing status in Chengdu	−1.306	1.683	−0.076	−0.776	−2.035	4.647	0.440
Income satisfaction	−3.747	1.383	−0.286	−2.709	−6.492	−1.001	0.008
Constant	27.027	12.591		2.147	2.039	52.016	0.034

Abbreviations are the same as in Table 2

## Discussion

In this survey, the majority of paid caregivers were married, middle-aged women with a low educational level. They suffered from a high caregiving burden that was related to the amount of time as a caregiver, income satisfaction and frequency of meeting with their own family. The depression rate of this group was higher than that of the general population, but it was lower than that of family caregivers; it was also positively correlated with caregiving burden.

### High caregiving burden in paid caregivers

The finding that paid caregivers suffered from a high caregiving burden was corroborated by the findings of previous reports; [21, 22] however, our study included a more diverse population of paid caregivers who worked in a hospital setting rather than a population of paid caregivers who worked for patients with a specific severe disease condition, such as dementia, cancer, and heart failure. Compared with family caregivers, paid caregivers showed a similar [23, 24] or higher [25, 26] CBI score.

The hired caregivers usually worked around the clock, except for short breaks at lunch and dinner or when the patients’ relatives came to visit. In particular, they always had to do everything by themselves at night, sleeping next to their patients in portable camping beds and observing the needs and movements of the patients; such sleep interruptions were frequent, inevitable and unpredictable. Although they could take a few vacation days per month, usually after fulfilling an existing contract and before starting a new one, they preferred not to do so because they were paid per working day. Therefore, this prolonged period of caregiving explains why the time-dependence burden was the most prominent among the 5 factors of the CBI; this finding was also observed in family caregivers. [16, 27, 28] Therefore, interventions to tackle this time-dependence burden could be suggested, e.g., caregiver companies could enforce a change from the current 24-h, 7-day working schedule to a better arrangement with day and night shifts. In addition, chronic sleep disturbances, tedious caregiving work and contact with infectious patients result in concerns about their own physical health, [12] which was the second most prominent burden among paid caregivers. This was different from that observed in family caregivers, who were more affected by a developmental burden than physical burden. [27, 29].

### Factors associated with a higher caregiving burden

Previous studies showed that the risk factors for caregiving burden included female gender, [30] a low education, [31, 32] sleep deprivation, financial stress, [33] depression, [34] social isolation, [35] and time restrictions [36]. Because the paid caregivers in the current study worked on a 24-h schedule, we gathered the number of years they spent as paid caregivers rather than the number of hours per day spent performing their job and found the former to be associated with the CBI score. This association might be attributed to the self-perceived long-term time restrictions and deteriorating physical health of the paid caregivers. As previously reported, financial stress was a risk factor for caregiving burden [33], and poor payment for paid caregivers was a common issue. [37, 38] In China, most tertiary referral hospitals (as the study site) provide high-quality, specialized medical health care services to a number of districts that are located in urban areas; however, these areas also have the largest demand for paid caregivers. [39] In contrast, most paid caregivers are from the countryside and have moved to a city for a better living. Not surprisingly, lower income satisfaction was significantly associated with a higher caregiving burden, indicating a conflict among their actual payment, self-expectations and financial circumstances. However, this conflict cannot be resolved only by raising their salaries. It is possible that organized lectures and regular meetings could identify what paid caregivers need and think. More interestingly, it was observed in the study that paid caregivers who met with their own families more frequently had a higher CBI score. This phenomenon has not been previously identified, and its cause remains unexplored in the current study.

### Correlation between depression and caregiving burden

Using the CES-D score ≥ 16 cut-off, we found that the prevalence of depression among paid caregivers reached 25.9%; this was higher than that of the general population in China, which was reported to be between 5.9% and 12.4%. [40, 41] However, other studies showed that more than 50% of family caregivers were at risk of depression according to the same CES-D score cut-off. [42, 43] This difference in the prevalence of depression might be explained by the “nature” of the caregiving work performed by paid caregivers compared to family caregivers. Paid caregivers usually consider the caregiving work as a job from which they are paid for their countable time and effort, while family caregivers treat it as a serious responsibility in which their emotions, time and money are involved. It is also possible that family caregivers are less likely to be gainfully employed. Consistent with previous studies on family caregivers [44, 45], our study showed that the caregiving burden of paid caregivers was positively correlated with depression symptoms.

### Study limitations

The major limitations of this study include its cross-sectional nature and the absence of health consequence data, i.e. the result of coupling between the caregiving burden and the physical/emotional health of paid caregivers. Although some practical interventions to reduce the caregiving burden could be developed based on the results of the current study, their impact on the professional health of paid caregivers remains unclear. The relatively small sample size is another limitation.

## Conclusions

Caregiving burden is high in paid caregivers of hospitalized patients in China, most of whom are middle-aged women from rural areas. It is associated with a prolonged working time, low income satisfaction and unexplained impact from their own families. In addition, the caregiver burden and depression are correlated, which is more prevalent in paid caregivers than in the general population.

## Abbreviations

CBI: Caregiver Burden Inventory
CES-D: Center for Epidemiologic Studies Depression Scale

## References

[1] Gillick MR (2013). The critical role of caregivers in achieving patient-centered care. JAMA.

[2] Polivka L (2005). The ethics and politics of caregiving. The Gerontologist.

[3] Gillick MR (2013). The critical role of caregivers in achieving patient-centered CareViewpoint. JAMA.

[4] Jorgensen D, Parsons M, Reid MG, Weidenbohm K, Parsons J, Jacobs S (2009). The providers' profile of the disability support workforce in New Zealand. Health Soc Care Community.

[5] Cho SH, Kim HR (2006). Family and paid caregivers of hospitalized patients in Korea. J Clin Nurs.

[6] Hui J, Wenqin Y, Yan G (2013). Family-paid caregivers in hospital health care in China. J Nurs Manag.

[7] Bevans M, Sternberg EM (2012). Caregiving burden, stress, and health effects among family caregivers of adult cancer patients. Jama-Journal of the American Medical Association.

[8] Adelman RD, Tmanova LL, Diana D, Sarah D, Lachs MS (2014). Caregiver burden: a clinical review. JAMA.

[9] Mi JM, Ling YX, Yang SQ (2008). The current situation of management of paid caregivers in hospitals. Nursing journal of Chinese people’s liberation army.

[10] Liu L, Hu XL, Li RM (2005). Analysis on status of accompanied nursing and proposals. Chin Nurs Res.

[11] Ling L, Zhang YJ, Sun HY (2007). Psychological health condition of professional caregivers and related factors. Chinese journal of practical nursing.

[12] Xu R, Li XY (2007). Investigation on pressure factors of full-time attendants in hospitals. Chin Nurs Res.

[13] Chou K-R, Jiann-Chyun L, Chu H (2002). The reliability and validity of the Chinese version of the caregiver burden inventory. Nurs Res.

[14] Liu PC, Gau BS, Hung CC. Development and Psychometric Testing of a Chinese Version of the Caregiver Burden Scale for Parents of Children With Allergies. J Pediatr Nurs. 2014; 10.1016/j.pedn.2014.04.006 24813162

[15] Novak M, Guest C (1989). Application of a multidimensional caregiver burden inventory. The Gerontologist.

[16] Zucchella C, Bartolo M, Pasotti C, Chiapella L, Sinforiani E (2012). Caregiver burden and coping in early-stage Alzheimer disease. Alzheimer Dis Assoc Disord.

[17] Rankin SH, Galbraith ME, Johnson S (1993). Reliability and validity data for a Chinese translation of the Center for Epidemiological Studies-Depression. Psychol Rep.

[18] Zhang J, Sun W, Kong Y, Wang C (2012). Reliability and validity of the Center for Epidemiological Studies Depression Scale in 2 special adult samples from rural China. Compr Psychiatry.

[19] Cheung CK, Bagley C (1998). Validating an American scale in Hong Kong: the Center for Epidemiological Studies Depression Scale (CES-D). Journal of Psychology Interdisciplinary & Applied.

[20] Radloff LS (1977). The CES-D scale a self-report depression scale for research in the general population. Appl Psychol Meas.

[21] Rosa E, Lussignoli G, Sabbatini F, Chiappa A, Di Cesare S, Lamanna L, Surrente B, Zanetti O (2008). The immigrant paid caregivers' role in the care of patients with severe dementia. International journal of geriatric psychiatry.

[22] Lin W-C, Tsai C-F, Wang S-J, Hwang J-P, Fuh J-L (2012). Comparison of the burdens of family caregivers and foreign paid caregivers of the individuals with dementia. Int Psychogeriatr.

[23] Raccichini A, Castellani S, Civerchia P, Fioravanti P, Scarpino O (2009). The caregiver's burden of Alzheimer patients: differences between live-in and non-live-in. American journal of Alzheimer's disease and other dementias.

[24] Patel M, Chawla R, Krynicki CR, Rankin P, Upthegrove R (2014). Health beliefs and carer burden in first episode psychosis. BMC psychiatry.

[25] Razani J, Kakos B, Orieta-Barbalace C, Wong JT, Casas R, Lu P, Alessi C, Josephson K (2007). Predicting caregiver burden from daily functional abilities of patients with mild dementia. J Am Geriatr Soc.

[26] Zhong M, Evans A, Peppard R, Velakoulis D (2013). Validity and reliability of the PDCB: a tool for the assessment of caregiver burden in Parkinson's disease. International psychogeriatrics / IPA.

[27] Pastore F (2014). Caregiver burden and coping strategies in caregivers of patients with alzheimer’s disease. Neuropsychiatr Dis Treat.

[28] Liu J, Wang LN, Tan JP, Ji P, Gauthier S, Zhang YL, Ma TX, Liu SN (2012). Burden, anxiety and depression in caregivers of veterans with dementia in Beijing. Arch Gerontol Geriatr.

[29] Moieni M, Poorpooneh Z, Pahlavanzadeh S (2014). Investigating the effect of family-focused nursing intervention on caregiver burden of the family members of the patients undergoing coronary bypass surgery in Isfahan Shahid Chamran hospital during 2012. Iranian journal of nursing and midwifery research.

[30] Gallicchio L, Siddiqi N, Langenberg P, Baumgarten M (2002). Gender differences in burden and depression among informal caregivers of demented elders in the community. International Journal of Geriatric Psychiatry.

[31] Vincent C, Desrosiers J, Landreville P, Demers L (2009). Burden of caregivers of people with stroke: evolution and predictors. Cerebrovasc Dis.

[32] Wong DFK, Lam AYK, Chan SK, Chan SF (2012). Quality of life of caregivers with relatives suffering from mental illness in Hong Kong: roles of caregiver characteristics, caregiving burdens, and satisfaction with psychiatric services. Health Qual Life Outcomes.

[33] Adelman RD, Tmanova LL, Delgado D, Dion S, Lachs MS (2014). Caregiver burden: a clinical review. JAMA.

[34] Gallagher D, Rose J, Rivera P, Lovett S, Thompson LW (1989). Prevalence of depression in family caregivers. The Gerontologist.

[35] Rodakowski J, Skidmore ER, Rogers JC, Schulz R (2012). Role of social support in predicting caregiver burden. Arch Phys Med Rehabil.

[36] Cole JC, Ito D, Chen YJ, Cheng R, Bolognese J, Li-McLeod J (2014). Impact of Alzheimer’s disease on caregiver questionnaire: internal consistency, convergent validity, and test-retest reliability of a new measure for assessing caregiver burden. Health Qual Life Outcomes.

[37] Fleming G, Taylor BJ (2007). Battle on the home care front: perceptions of home care workers of factors influencing staff retention in Northern Ireland. Health & Social Care in the Community.

[38] Banaszak-Holl J, Hines MA (1996). Factors associated with nursing home staff turnover. The Gerontologist.

[39] Xiao LD (2010). Continuing nursing education policy in China and its impact on health equity. Nurs Inq.

[40] Yang L, Jia CX, Qin P (2015). Reliability and validity of the Center for Epidemiologic Studies Depression Scale (CES-D) among suicide attempters and comparison residents in rural China. BMC Psychiatry.

[41] Hou F, Cerulli C, Wittink MN, Caine ED, Qiu P (2015). Depression, social support and associated factors among women living in rural China: a cross-sectional study. BMC Womens Health.

[42] Lv Y, Zhao Q, Li X, Stanton B, Fang X, Lin X, Zhao G, Zhao J (2010). Depression symptoms among caregivers of children in HIV-affected families in rural China. Aids Care-Psychological and Socio-Medical Aspects of Aids/Hiv.

[43] Eisdorfer C. Depression and family caregiving: findings from the Miami site of the resources for enhancing Alzheimer's caregiver health (REACH) program. Int Psychogeriatr. 2003;160

[44] Oh J, An JW, Oh K-W, Oh S-I, Kim JA, Kim SH, Lee JS (2015). Depression and caregiving burden in families of patients with amyotrophic lateral sclerosis. J Korean Acad Nurs.

[45] Chung ML, Lennie TA, Mudd-Martin G, Dunbar SB, Pressler SJ, Moser DK (2016). Depressive symptoms in patients with heart failure negatively affect family caregiver outcomes and quality of life. Eur J Cardiovasc Nurs.

